# A Survey on the Use of Antibiotics among the Dentists of Kolkata, West Bengal, India

**DOI:** 10.5005/jp-journals-10005-1497

**Published:** 2018-04-01

**Authors:** Rahul Kaul, Paras Angrish, Parul Jain, Subrata Saha, Ashok V Sengupta, Shantanu Mukherjee

**Affiliations:** 1 Postgraduate Student, Department of Pedodontics and Preventive Dentistry Dr. R Ahmed Dental College and Hospital, Kolkata, West Bengal, India; 2 Postgraduate Student, Department of Pedodontics and Preventive Dentistry Dr. R Ahmed Dental College and Hospital, Kolkata, West Bengal, India; 3 Postgraduate Student, Department of Pedodontics and Preventive Dentistry Dr. R Ahmed Dental College and Hospital, Kolkata, West Bengal, India; 4 Professor, Department of Pedodontics and Preventive Dentistry Dr. R Ahmed Dental College and Hospital, Kolkata, West Bengal, India; 5 Associate Professor, Department of Pedodontics and Preventive Dentistry Dr. R Ahmed Dental College and Hospital, Kolkata, West Bengal, India; 6 Associate Professor, Department of Pedodontics and Preventive Dentistry Dr. R Ahmed Dental College and Hospital, Kolkata, West Bengal, India

**Keywords:** Analgesics, Antibiotics, Overuse, Resistance.

## Abstract

**Introduction:**

Dental infections are multimicrobial in origin with their etiological factors involving a combination of Gram-positive, Gram-negative, facultative anaerobes, and obligate anaerobic bacteria. Thus, antibiotics and analgesics account for a great majority of medicines prescribed by the dental surgeons. Inappropriate prescription of antibiotics by health care professionals has become a worldwide issue nowadays.

**Aim:**

The purpose of the present study was to:

• Determine the pattern of antibiotic prescription among dentists of Kolkata.

• Assess the attitude of dentists toward antibiotic resistance.

**Materials and methods:**

An electronic version of the questionnaire of cross-sectional survey regarding antibiotic use and attitude toward growing antibiotic resistance was constructed using Qualtrics (Qualtrics Pvt. Limited Provo, Utah), an internet online survey tool that was e-mailed to dental surgeons of Kolkata registered with Indian Dental Association (IDA), West Bengal. A reminder e-mail was given after 1 month to recollect the responses from them.

**Results:**

The survey was e-mailed to 300 dental surgeons, out of which 115 dental surgeons completed the survey, thereby achieving a response rate of 38.33%. Majority of the respondents (60%) chose amoxicillin in nonallergic patients. Average minimum duration of antibiotic therapy was 5 days. The drug of first choice for patients with an allergy to penicillin was erythromycin. The prime determinant of antibiotic use was facial swelling (68%). The prime determinant to select a particular brand of antibiotics was affordability of that brand (61%). Almost all (99%) dental surgeons were aware of antibiotic resistance being a growing concern. As per their views, there was overprescription of antibiotics.

**How to cite this article:** Kaul R, Angrish P, Jain P, Saha S, Sengupta AV, Mukherjee S. A Survey on the Use of Antibiotics among the Dentists of Kolkata, West Bengal, India. Int J Clin Pediatr Dent 2018;11(2):122-127.

## INTRODUCTION

Chemotherapy has been defined as the use of synthetic, semi-synthetic, and naturally occurring chemicals that inhibit specific organisms causing disease. The term “antibiotic” has been derived from combination of two words: Anti meaning “against” and biosis meaning “life.” Proper use of antibiotics along with surgical therapy is the most appropriate method to treat various odontogenic infections. Alexander Fleming, who along with Howard Florey and Ernst Chain shared the Nobel Prize in 1945 in Physiology and Medicine, addressed in his lecture: “It is not difficult to make microbes resistant to penicillin in the laboratory by exposing them to concentrations not sufficient to kill them, and the same thing has occasionally happened in the body.”^1^ Prescribing antibiotics by dental practitioners has become an important aspect of day-today dental practice. This is the reason why antibiotics account for a huge majority of medicines being prescribed by dentists. It has been observed that contribution toward the problem of antibiotic resistance by dentists can be substantial as dentists prescribe 10% of all common antibiotics.^2^ Yingling et al^3^ concluded from findings of his study among members of the American Association of Endodontists (AAE) that they were prescribing antibiotics inappropriately. On the contrary, the National Center for Disease Control and Prevention found that almost one-third of all outpatient antibiotic prescriptions are unnecessary.^4^ We have now entered an era where some bacterial species are resistant to the full range of antibiotics presently available, with the methicillin-resistant *Staphylococcus aureus* and vancomycin-resistant *Staphylococcus aureus* being the most widely known example of extensive resistance.^5^ Keeping in mind the results obtained from previous studies, we took up the present study to investigate antibiotic prescribing practices of dentists. The aim of the present study was to determine the pattern of antibiotic prescription among dental surgeons of Kolkata and assess their attitude toward growing antibiotic resistance.

## MATERIALS AND METHODS

The present study was designed as a descriptive cross-sectional study performed among the dentists of Kolkata registered under the IDA West Bengal Branch after obtaining ethical clearance from Ethics Committee, Dr. R Ahmed Dental College and Hospital, Kolkata, West Bengal, India. The study had a sample size of 300 dentists (Table 1). Inclusion criteria were any dental practitioner holding the Bachelor of Dental Surgery (BDS) and Master of Dental Surgery (MDS) degree registered with IDA West Bengal branch who practiced within the limits of municipal committee, Kolkata city. The exclusion criteria included dentists registered with IDA West Bengal practicing outside Kolkata. An electronic version of pretested and prevalidated questionnaire consisting of both open-ended and close-ended questions (Table 2) of the cross-sectional survey regarding antibiotic use and resistance was constructed using Qualtrics (Qualtrics Pvt. Limited Provo, Utah), an internet online survey tool. A link was generated that was e-mailed to dentists of Kolkata. A reminder e-mail was given after 1 month. Study was conducted over a period of 2 months. The data were statistically analyzed. Data analysis included descriptive and analytical statistics.

**Table 1:** **Table 1:** Demographic characteristics

*Variable*		*Number of dentists (%)*	
*Sex*			
Male		61	
Female		39	
*Age*			
Less than 30 years		71	
More than 30 years		29	
*Years since practicing dentistry*			
<5		48	
5-10		37	
10-15		9	
>15		6	
*Educational qualification*			
BDS		22	
MDS		78	
*Practice type*			
Private practice		29	
Academic institution		55	
Hospital dentistry		34	
Health center/any trust		3	

## RESULTS

Present survey link was e-mailed to 300 dentists registered with IDA, West Bengal Branch, out of which 115 dentists completed the survey, thereby achieving a response rate of 38.33%. Responders of present survey included 62% males and 38% females with 71% respondents being <30 years of age; 51% respondents had run a practice of >5 years. Majority of the respondents were specialists, while a lesser number were general practitioners (23% BDS and 77% MDS). The main source of updated information regarding the use of antibiotics was found to be the latest editions of textbooks (57%). Most of the respondents (60%) chose amoxicillin in nonallergic patients [alone (37%) or associated with clavulanic acid (23%)]. Amoxi-cillin was prescribed as the first-choice antibiotic by 37% of respondents, which is appropriate for oral infection, while 34, 23, and 2% selected a combination of amoxicil-lin and metronidazole, amoxicillin/clavulanic acid, and ofloxacin + ornidazole respectively (Table 3).

**Table 2:** **Table 2:** Questionnaire

*Demographic information and practice characteristics*			
*Gender:*			
• Male			
• Female			
*Age:*			
• <30 years			
• >30 years			
*Years since practicing dentistry:*			
• <5			
• 5-10			
• 10-15			
• >10			
*Practice type:*			
• Private practice			
• Academic institution			
• Hospital dentistry			
• Health center/any trust			
Specialization:...............................................................			
*Kindly mark the suitable option:*			
(1) What is your primary source of updated information?			
• Scientifically published literature			
• Continuing dental education (CDE) and conferences			
• Textbooks (latest editions)			
• Internet			
(2) Most commonly prescribed antibiotic:			
• Amoxicillin			
• Amoxiclav			
• Amoxicillin + Metronidazole			
• Ofloxacin + Ornidazole			
(3) If a patient is allergic to penicillin, which antibiotic do you usually prescribe?			
• Erythromycin			
• Clindamycin			
• Azithromycin			
• Others			
(4) Most common route of administration:			
• Oral			
• Intravenous (5) Minimum number of days for prescribing antibiotic:			
• 3			
• 5			
• 7			
• 10			
(6) Most common determinant for prescribing antibiotics:			
• Facial swelling			
• Pain relief			
• Unavailable appointment for several weeks			
• Patient satisfaction/parent satisfaction (pedo patients)			
• Prophylactic (before extraction)			
(7) Which of the following factors do you consider primarily while prescribing particular brand of antibiotics?			
• Popularity of the brand			
• Availability of the brand			
• Affordability of the brand			
(8) In which of the following pulpal and periradicular condition do you prescribe antibiotics?			
• Reversible pulpitis			
• Irreversible pulpitis			
• Localized dentoalveolar abscess			
• Localized dentoalveolar abscess with draining fistula			
• Facial cellulitis			
(9) In which of the following conditions would you prescribe antibiotics in case of dental trauma?			
• Replantation after avulsion			
• Intrusion			
• Extrusion			
• Lateral luxation			
• Subluxation			
• Noncontaminated dental injuries			
(10) Mention the conditions under which you give infective endocarditis prophylaxis:			
• Previous infective endocarditis			
• Cardiac transplant after valvular damage			
• Cyanotic heart disease			
• Mitral valve prolapse with regurgitation			
• Mitral valve prolapse without regurgitation			
• Rheumatoid arthritis			
(11) Do you believe that antibiotic resistance is of growing concern?			
• Yes			
• No			

If patient was found to be allergic to penicillin, drug of first choice in that case was erythromycin (53%), followed by azithromycin (22%) and clindamycin (19%). As per the results of the current study, the minimum duration of antibiotic therapy was 5 days for majority (59%), while a considerable number of respondents prescribed antibiotics for a minimum of 3 days (35%). Principal determinant for prescribing antibiotics was facial swelling (68%). Affordability of that particular brand (61%) was the factor that was primarily considered while prescribing a particular brand. Overall, evidence suggesting over prescription was found for the following conditions: reversible pulpitis (13%), irreversible pulpitis (Table 4) with and without vital pulp (35%), localized dentoalveolar abscess with (57%) and without draining fistula (50%) (Table 5), mitral valve prolapse with regurgitation (43%), intrusion of tooth (25%), extrusion of that tooth (36%), lateral luxation (32%), subluxation (17%), and rheumatoid arthritis (16%). Almost everyone (99%) among the respondents believed that antibiotic resistance is of growing concern. Being aware of the fact that there was emerging problem of drug resistance, there was overprescription of antibiotics.

**Table 3:** **Table 3:** Responses to the questionnaire

*Question*			*Response (n)*			
1.	Primary source of updated information:					
	Scientifically published literature		52		45	
	CDE programs and conferences		19		16	
	Textbooks (latest edition)		66		57	
	Internet		56		48	
2.	Most commonly prescribed antibiotic:					
	Amoxicillin		43		37	
	Amoxiclav		27		23	
	Amoxicillin and metronidazole		40		34	
	Ofloxacin and ornidazole		2		2	
	Cephalexin		0		0	
	Others		3		3	
3.	If a patient is allergic to penicillin which antibiotic do you usually prescribe?					
	Erythromycin		61		53	
	Clindamycin		22		19	
	Azithromycin		26		22	
	Others		6		6	
4.	Most common route of administration:					
	Oral		114		99	
	Intravenous		1		1	
5.	Minimum number of days for prescribing antibiotic:					
	3		41		35	
	5		67		59	
	7		7		6	
	10		0		0	
6.	Most common determinant for prescribing antibiotics:					
	Facial swelling		77		68	
	Pain relief		47		42	
	Unavailable appointment for several weeks		10		9	
	Patient satisfaction		4		4	
	Parent satisfaction (pedo patient)		5		4	
	Prophylactic (as before extraction)		55		49	
7.	Factors you consider primarily while prescribing particular brand of antibiotics:					
	Popularity of brand		31		27	
	Availability of brand		58		50	
	Affordability of brand		70		61	
8.	In which of the following pulpal and periradicular condition do you prescribe antibiotics?					
	Reversible pulpitis		14		13	
	Irreversible pulpitis		39		35	
	Localized dentoalveolar abscess		56		50	
	Localized dentoalveolar abscess with draining fistula		63		57	
	Facial cellulitis		78		70	
9.	In which of the following conditions would you prescribe antibiotics in case of dental trauma?					
	Replantation after avulsion		95		89	
	Intrusion		27		25	
	Extrusion		38		36	
	Lateral luxation		34		32	
	Subluxation		18		17	
	Noncontaminated dental injuries		24		22	
10.	Mention the conditions under which you give infective endocarditis prophylaxis:					
	Previous infective endocarditis		101		89	
	Cardiac transplant after valvular damage		64		57	
	Cyanotic heart disease		38		34	
	Mitral valve prolapse with regurgitation		59		52	
	Mitral valve prolapse without regurgitation		11		10	
	Rheumatoid arthritis		18		16	
11.	Whether antibiotic resistance is of growing concern?					
	Yes		114		99	
	No		1		1	

## DISCUSSION

The decision to use an antimicrobial/antibiotic agent in managing an odontogenic infection is based on several factors. Clinician must first diagnose the cause of the infection and then determine the appropriate dental treatment that may include multiple modalities, including initiation of endodontic therapy and pulpectomy, mechanical or surgical disruption of that infectious environment. Before coming to a decision whether we require conjunctive antibiotic therapy, various factors, including host defense mechanisms, severity of the infection, magnitude of the extension of the infection, and expected pathogen, have to be taken into consideration. The fact that there is absence of collateral circulation within dental pulp makes the normal host defenses (inflammation and immunity) compromised, thereby making root canal system a unique environment to harbor a limited group of bacteria. The effectiveness of an oral antibiotic as first choice of treatment for an infection of odontogenic etiology is highly questionable as there is a lack of effective circulation in a necrotic pulp system. This concept reinforces the idea that surgery of some kind is the primary treatment of an infection of odontogenic source and that antibiotic therapy is simply an adjunctive care.^4^

The present study evaluated the antibiotic prescribing practices among the dental surgeons of Kolkata and their attitude toward growing concern of antibiotic resistance. In the present study, the questions were based on those in previous surveys developed in the USA^3^ and Spain.^6,7^ The overall response rate was 38.3%, and similar response rates were found in other published surveys.^8,9^

Endodontic infections typically have a rapid onset and short duration, most specifically if the cause is treated or eliminated. Prolonged courses of antibiotics destroy the commensal flora, thereby abolishing colonization resistance. The prescribing of systemic antibiotics hence needs to be justified. Broad spectrum of activity of amoxicillin, what is required for endodontic needs and its injudicious use in a healthy individual may contribute to the global antibiotic resistance problem.^10^

The results of the present study were in accordance to the results obtained from a study conducted in the USA and found that amoxicillin was prescribed only by 27.5% of members of the AAE.^3,11^ This study found that the second most frequently prescribed antibiotic for non-penicillin-allergic patients was a combination of amoxicil-lin and metronidazole (34%). On the contrary, as per the survey conducted in Spain, the leading antibiotic was amoxicillin plus clavulanic acid, followed by amoxicillin alone.^12^ Amoxicillin has also been found to be the most commonly prescribed antibiotic in European countries.^13^ In the present study, the drug of first choice in patients with an allergy to penicillins was found to be erythromycin (53%). In contrast to the results of the present study, clindamycin was the most prescribed drug in penicillin-allergic patients in the USA (21.6 and 57.03%)^3,11^ and Spain (63.2 and 65.4%).^6,7^ Other antibiotics prescribed for patients with an allergy to penicillins were azithromycin (22%) and clindamycin (19%).

**Table 4:** **Table 4:** Multivariate logistic regression analysis of the influence of the independent variables [gender (male: 1, female: 2), age (1: <30 years, 2: >30 years), experience (1: <10 years, 2: >10 years), and academic degree (1: BDS, 2: MDS)] on the dependent variable antibiotic prescription for irreversible pulpitis (0: No, 1: Yes)

*Independent variable*		*B*		*p-value*		*Odds ratio*		*95% confidence interval*	
Gender		0.594		0.03*		2.473		1.219-2.870	
Age		0.729		<0.001*		3.194		1.539-3.816	
Experience		0.714		<0.001*		3.0831		1.891-3.571	
Academic qualification		0.804		<0.001*		3.55		2.471-3.906	

*Statistically significant at p-value < 0.05

**Table 5:** **Table 5:** Multivariate logistic regression analysis of the influence of the independent variables [gender (male: 1, female: 2), age (1: <30 years, 2: >30 years), experience (1: <10 years, 2: >10 years), and academic degree (1: BDS, 2: MDS)] on the dependent variable antibiotic prescription for localized dental abscess (0: No, 1: Yes)

*Independent variable*		*B*		*p-value*		*Odds ratio*		*95% confidence interval*	
Gender		0.519		0.048*		1.947		0.351-2.804	
Age		0.619		0.602		1.0836		0.473-1.984	
Experience		0.308		0.709		–1.004		–0.003 to –1.1572	
Academic qualification		0.614		0.043*		2.503		1.845-2.765	

*Statistically significant at p-value < 0.05

Azithromycin does not find any role in oral infection because about 82% of oral streptococci develop resistance to macrolides after a single course.^14^ Clindamycin is a good choice if the patient is allergic to penicillin, although clindamycin has a low, but serious, risk of pseudomem-branous colitis.^15^

Metronidazole has been found to be very effective against obligate anaerobes, but not against facultative anaerobic bacteria. Hence, it becomes necessary for it to be used in conjunction with other agents. Moreover, if within 48 hours the patient is not responding to penicillin alone, one can consider adding metronidazole to the existing drug regimen.

Inadequate duration of the therapy or overdosing of the antibiotics has resulted in damaging the host response, thereby producing toxic effects.^3^ Treatment of most odontogenic infections requires an average of 5 to 7 days of therapy; however, treatment of severe infections or immunocompromised patients’ therapy can be of longer duration owing to reduced immunity. A rule of thumb when prescribing is that the antibiotic should last for 3 days after the patient’s symptoms have been resolved.^3^

In the present study, many respondents (35%) responded that they will give antibiotics for a minimum of 3 days. Patient compliance also plays a major role in effective treatment. Most of the patients stop the drug therapy as and when initial symptoms are resolved. Affordability of brand was considered main factor while prescribing a particular brand. The drug may be too expensive or not covered by a third-party payer and the prescription remains unfilled. An alternative to this can be prescribing generic drugs.

Dosing frequency may be complicated. The compliance issue most often observed is missed doses after clinical symptoms have subsided. Another challenge to compliance is the untoward or unexpected side effects that can occur when taking antibiotics. In all these cases, there is a chance for mutated microbes to flourish and thereby causing serious consequences.

Endodontic conditions like reversible pulpitis, irreversible pulpitis with moderate/severe preoperative symptoms, with or without acute periodontitis do not warrant antibiotic coverage. Despite these facts, we found in our present study that 35% respondents prescribed antibiotics prior to beginning of treatment. This finding is slightly higher than those reported in previous studies.^3,16-18^

Necrotic pulp with dentoalveolar abscess, with or without fistula, with no swelling, and no/mild symptoms does not require use of antibiotics as treatment can be restricted to nonsurgical root canal therapy; however, 56% respondents of this study population reported antibiotic use. In the present study, a higher percentage of dentists prescribed antibiotics for necrotic pulp with chronic apical periodontitis with sinus tract and no/mild preoperative symptoms as compared with that prescribed by Spanish^6^ and American dentists.^3^ Rational use of antibiotics is based on well-defined indications in cases of infection of endodontic origin.

Therefore, antibiotics must be considered only as an adjunct to conventional root canal therapy or when emergency treatment is not possible.^19,20^ This outcome suggests that the scientific basis for prescribing antibiotics was ignored by the majority of the dental surgeons. Dental surgeons need to have a thorough understanding of the clinical indications for antibiotic prescription in order to prevent the misuse or overuse of these drugs.^20^ Since the majority of respondents were young, i.e., age <30 years, it is their duty toward society to initiative not to repeat these practices, instead help in prevention of resistance problem which is now accepted as a challenge worldwide.

## STUDY LIMITATIONS

We need to consider the results of this study in light of some study limitations. Since the survey was self-administered, responses may have been subject to response bias. The dentists who participated may not be the representative of dentists of Kolkata. Although a few trends were evident, the sample size was small and thus inferences were difficult.

Despite all these limitations, this study has several strengths, including being the first in the region to our knowledge to report on this topic of importance and clinical relevance. The study results provide preliminary data regarding extent to which professionals were adhering to professional guidelines. The data also included patterns among not only general dentists but also all specialties. The present study sets the stage for further research.

## CONCLUSION

Dental surgeons in Kolkata were found to overprescribe antibiotics, which could be a major contributor to the worldwide problem of antimicrobial resistance. Most of the respondents of this survey were young dentists. It is their responsibility to curb this problem instead of going along with the flow. There is an urgent need to raise public and professional awareness regarding the risks of injudicious use of antibiotics in dentistry.
